# MicroRNAs in Systemic Sclerosis: Involvement in Disease Pathogenesis and Potential Use as Diagnostic Biomarkers and Therapeutic Targets

**DOI:** 10.3390/biomedicines13051216

**Published:** 2025-05-16

**Authors:** Russka Shumnalieva, Simeon Monov, Tsvetelina Velikova

**Affiliations:** 1 Department of Rheumatology, Clinic of Rheumatology, University Hospital “St. Anna” Sofia, Medical University-Sofia, Dimitar Mollov Street 1, 1709 Sofia, Bulgaria; doctor_monov@yahoo.com; 2 Medical Faculty, Sofia University St. Kliment Ohridski, 1 Kozyak Street, 1407 Sofia, Bulgaria; tsvelikova@medfac.mu-sofia.bg

**Keywords:** systemic sclerosis, autoimmune, microangiopathy, extracellular matrix, epigenetic, miRNA, biomarkers, therapeutic targets

## Abstract

Systemic sclerosis (SSc) is a chronic autoimmune connective tissue disorder characterized by three main pathological features: microangiopathy, immunological alterations, and excessive synthesis of extracellular matrix (ECM) proteins, leading to fibrosis of the skin and internal organs. Although the etiology of SSc is still unknown, recent studies have revealed the potential role of genetic and epigenetic factors in disease pathogenesis. They are involved in the regulation of cell metabolism, cell hyperactivity, and the accumulation of extracellular matrix proteins. Short endogenous noncoding RNA molecules (microRNAs; miRNAs) negatively regulate gene expression at the posttranscriptional level and play a significant role in disease pathogenesis. Altered miRNA expression in circulation and disease-specific tissues could serve as biomarkers and potential therapeutic targets in SSc.

## 1. Introduction

Systemic sclerosis (SSc) is a chronic autoimmune connective tissue disorder defined by three major pathological features—microangiopathy, immunological alterations, and excessive synthesis of extracellular matrix (ECM) proteins—leading to fibrosis of the skin and internal organs [1]. The clinical presentation differs among patients and is divided into limited or diffuse SSc, defined by the extent of skin fibrosis. Among the known risk factors for the disease are some toxic agents, longstanding Raynaud’s phenomenon, or tissue injuries [2,3]. The pathophysiology of SSc is characterized by progressive endothelial damage, the dysregulation of innate immunity, and dysfunctional angiogenesis, affecting the microvasculature due to impaired differentiation ability of endothelial progenitor cells (EPCs) and their interplay with angiogenic factors like vascular endothelial growth factor (VEGF) [4,5]. There is increased adhesion of peripheral monocytes, macrophage polarization toward M2 phenotype with profibrotic properties, and a macrophage-to-myofibroblast transition in the skin and affected organs in SSc [6]. One of the key cytokines in the pathogenesis of SSc is the transforming growth factor (TGF), which plays a pivotal role in regulating the fibrotic process through multiple signaling pathways [7].

Although the etiology of SSc is still unknown, recent studies reveal the probable role of genetic and epigenetic factors in the regulation of cell metabolism, hyperactivity, and the accumulation of collagen and other ECM proteins in the tissues [8]. Of potential biological significance for the development of SSc are the epigenetic modifications of gene expression through reversible changes in chromatin structures, including DNA methylation, histone modification, and miRNA expression [9,10,11].

## 2. miRNA Overview

MicroRNAs (miRNAs) are small, naturally occurring non-coding RNA molecules that regulate gene expression post-transcriptionally by binding to messenger RNAs (mRNAs) and inhibiting their translation or promoting their degradation [12]. Experimental studies have revealed the role of miRNA in the regulation of essential fibrosis-related signaling pathways and molecules involved in the cell hyperactivity state and the pathogenesis of tissue fibrosis. There is a deregulated expression of miRNAs both in the circulation and tissues specifically affected by the disease, and miRNA expression in SSc patients differs significantly from that observed in healthy controls, where miRNA alterations correlate with the disease itself, disease manifestations, or immunological alterations. Due to their stability and role in regulating immune response and cytokine production, miRNAs have been widely explored as potential diagnostic and activity biomarkers in SSc, as well as potential treatment targets [13,14,15,16,17].

### miRNA Biosynthesis

MiRNA biosynthesis is classified into canonical and non-canonical pathways.

The canonical pathway is the main pathway for miRNA biosynthesis and includes the formation of the pri-miRNA transcript, which is subsequently cleaved by the Drosha–DGCR8 (DiGeorge Syndrome Critical Region 8) complex into a precursor miRNA (pre-miRNA), which is then transported to the cytoplasm by the Exportin-5/RanGTP complex. In the cytoplasm, pre-miRNA undergoes further processing into a miRNA duplex by Dicer and a co-factor protein—the transactivation response RNA Binding Protein. One strand of the miRNA duplex is selectively incorporated into a member of the Argonaute (AGO) protein family, forming the core of the miRNA-induced silencing complex (miRISC). miRISC binds to target mRNA to inhibit translation via nine possible mechanisms:

- Suppression of chain elongation.
- mRNA cleavage.
- mRNA destabilization.
- Suppression of the binding of the 60S subunit of the ribosomes.
- Suppression of the binding of the 40S subunit of the ribosomes.
- miRNA induces reorganization of the chromatin and blocks gene expression.
- Premature ending of translation.
- Cleavage of co-translational proteins.
- Sequestration of P-bodies* [18,19,20,21,22].

**Figure 1:**
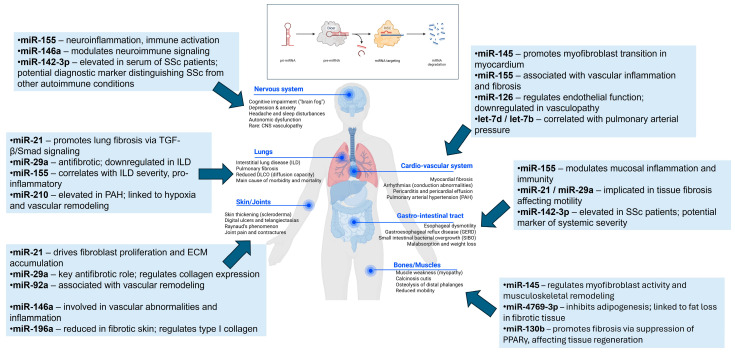
Mechanisms of microRNA-mediated regulation in systemic sclerosis pathogenesis. The organ-specific involvement of microRNAs (miRNAs) in systemic sclerosis (SSc) highlights their roles in key pathological processes such as fibrosis, immune dysregulation, and vascular remodeling. The listed miRNAs represent potential diagnostic biomarkers and therapeutic targets relevant to the clinical manifestations of SSc in the nervous, cardiovascular, pulmonary, gastrointestinal, musculoskeletal, and cutaneous systems. Created in BioRender. Velikova, T. (2025) https://BioRender.com/8j067yv; https://BioRender.com/5dex6kl and PowerPoint.

miRNA can be synthesized from small hairpin RNAs (shRNA), mirtrons, or 7-methylguanine-capped (m7G)-pre-miRNA through non-canonical pathways. The shRNAs are processed in the nucleus and exported by Exportin5/RanGTP transport protein, but are further processed in the cytoplasm in an AGO2-dependent but Dicer-independent manner. In contrast, both mirtrons and 7-methylguanosine-capped (m7G) pre-miRNAs undergo Dicer-dependent maturation in the cytoplasm, but they utilize distinct pathways for nuclear export: mirtrons are transported by Exportin-5/RanGTP, whereas m7G-capped pre-miRNAs are exported via Exportin-1 [23,24]. The pathways of miRNA regulation and function in connection with SSc pathogenesis are presented in Figure 1.

## 3. miRNA Alterations in SSc

It has been shown that miRNAs influence the fibrotic process in SSc in several ways—by targeting signaling pathways, including the transforming growth factor (TGF)/Smad3 canonical pathway, along with the connective tissue growth factor (CTGF) and NOTCH signaling pathways; contributing to the regulation of the epithelial-to-mesenchymal transition (EMT); promoting myofibroblast proliferation; and enhancing resistance to apoptosis [10,25,26]. Alterations in miRNA expression have been found in circulation and tissue-specific cells in SSc where, for example, miR-181a was downregulated, miR-132, miR-143, miR-145, and miR-155 were overexpressed in the serum, and miR-4769 was upregulated in the plasma and skin lesions of SSc patients [27,28,29]. Dividing miRNAs in SSc into profibrotic or antifibrotic depends on their properties to induce or suppress the fibrotic process in vitro and in vivo.

### 3.1. miRNA Alterations Related to the Pathogenesis of Fibrosis in SSc Patients

#### 3.1.1. miR-29

The miR-29 family comprises of miR-29a, miR-29b-1, miR-29b-2, and miR-29c. Because of its pivotal role in the pathogenesis of fibrosis, it is known as “master fibromiRNA”. miR-29 has antifibrotic effects by targeting fibrosis-related genes, both structural ECM and enzymes, which are involved in tissue remodeling, including collagens, fibronectin, laminin, and matrix metalloproteinase-2 (MMP-2). Functional studies have demonstrated that miR-29 inhibits the TGF-β1/Smad signaling cascade, thereby attenuating the pro-fibrotic effects induced by TGF-β1. Specifically, miR-29 targets TGF-β-activated kinase 1 binding protein 1 (TAB1), leading to the reduced expression of tissue inhibitor of metalloproteinases 1 (TIMP-1) in dermal fibroblasts. The expression of miR-29 is commonly downregulated across various fibrotic conditions, with levels showing an inverse correlation with fibrosis severity. Moreover, the miR-29 family exhibits dual roles in tumor biology, functioning either as a promoter or suppressor of tumorigenesis through its regulation of both pro-apoptotic and anti-apoptotic Bcl-2 family members. Notably, miR-29 can modulate the expression of Mcl-1, an anti-apoptotic Bcl-2 protein, thereby influencing cell survival and apoptosis pathways [30,31,32].

miR-29 serves as a key regulator of collagen expression in SSc by directly suppressing the synthesis of collagen types I, II, and IV. It has been shown that miR-29 induces apoptosis in dermal fibroblasts from SSc patients by increasing the Bax–Bcl2 ratio. A marked decrease in miR-29a expression has been observed in the fibroblasts and skin tissues of SSc patients compared to healthy controls by Maurer et al. [33]. In dermal fibroblasts of diffuse cutaneous SSc patients and in fibroblasts treated with TGF-β, miR-21 expression was elevated while miR-29a was suppressed, reflecting the expression patterns observed in our previous studies [34,35].

Changes in miR-29a expression have also been detected in hair samples of SSc patients compared to healthy controls. Interestingly, Wajda et al. reported that serum levels of miR-29a were significantly elevated only in patients with limited cutaneous SSc (lSSc) relative to healthy controls [27]. Kawashita et al. found an association between miR-29a downregulation in the serum and increased right ventricular systolic pressure in SSc patients [36]. Moreover, Luo et al. demonstrated that hypoxia-induced activation of pulmonary adventitial fibroblasts is linked to the reduced expression of miR-29a-3p, implying a regulatory role for this miRNA in cellular activation, proliferation, and secretory functions, thus serving as a potential therapeutic target in hypoxic pulmonary hypertension [37].

The altered miRNA expression in SSc was found to correlate with immunological parameters. Wuttge et al. showed that plasma levels of miR-29a vary between patients with lSSc who are positive for anticentromere antibodies (ACA) and those with anti-U1 ribonucleoprotein (RNP) antibodies [38]. In our previous study, we found a good correlation between miR-29a serum levels and the presence of anti-Scl70 antibodies, as well as with the serum levels of miR-21 [34].

#### 3.1.2. miR-27a-3p

miR-27 has been classified as an oncogenic miRNA due to its role in regulating transcription factors involved in tumor cell survival and proliferation [38,39,40]. It also serves as an activator of the Wnt-signaling pathway and promotes osteoblast differentiation [41]. By targeting similar signaling pathways, miR-27a is known to play a role in the pathogenesis of cancer, as well as several fibrotic processes, including idiopathic pulmonary fibrosis, renal fibrosis, and cardiac and skin fibrosis [42,43,44]. It has been found that miR-27 modulates the synthesis of ECM proteins, particularly collagen type I, by targeting the gremlin 1 protein [45,46]. The overexpression of miR-27a-3p is related to the downregulation of fibrosis-related genes. It was shown that miR-27a-3p regulates the secreted phosphoprotein 1 (SPP1) expression in the miR-27a-3p-SPP1-ERK1/2 regulatory axis during transformation of the myofibroblasts and could suppress lung and skin fibrosis in SSc patients [47]. Zeng et al. identified TGFβ receptor 1 and Smad2 as targets of miR-27b and suggested that miR-27 has antifibrotic properties in models of pulmonary fibrosis [48]. Another aspect of miR-27 biology is its role in regulating adipogenesis. MiR-27a inhibits peroxisome proliferator-activated receptor gamma (PPARγ) expression at the posttranscriptional level and thus acts as a negative control in adipocyte differentiation. The link between PPARγ signaling and miR-27 expression suggests that miR-27 exhibits an antifibrotic effect in SSc by regulating TGFβ signaling [49]. Bayati et al. found significant downregulation of miR-27 expression levels in whole-blood samples from SSc patients compared to healthy controls, as well as in patients positive for anti-topoisomerase (ATA) antibodies compared to the negative ones [50].

#### 3.1.3. miR-21

The most studied miRNA in SSc is miR-21. miR-21 is a widely conserved microRNA that is broadly expressed in various cell types and is best known for its involvement in tumor development and progression, i.e., facilitates the epithelial–mesenchymal transition (EMT) and autoimmunity. miR-21-5p has been mapped at 17q23.2, overlapping with the gene for vacuole membrane protein 1 (VMP1) [51,52]. miR-21 is upregulated by TGF-β1, which in turn induces TGF-β1-related fibrogenesis in skin fibroblasts through targeting Smad7. The latter has been found to be a direct target of miR-21. The expression of miR-21 has been found to be increased in SSc skin tissues and fibroblasts, promoting the fibrotic process by stimulating fibroblast proliferation and enhancing the deposition of extracellular matrix (ECM) components [53]. Together with miR-31 and miR-155, it participates in SSc-related vasculopathy and fibroproliferative alterations [54].

An in vivo bleomycin-induced SSc murine model that investigated the effects of miR-21 expression and inhibition was employed by Park et al. [55]. They used C57BL/6 mice, in which fibrosis was induced by infecting bleomycin. It was demonstrated that miR-21 promoted lung and skin fibrosis by increasing the infiltration of cells secreting TNF-α, IL-1β, IL-6, and IL-17. In contrast, administering anti-miR-21 led to reduced infiltration and production of inflammatory cytokines. These results pave the way for further investigation of miR-21 inhibition as a therapeutic approach for SSc-associated fibrosis [55].

Zhu et al. performed a series of studies regarding different miRNAs and cytokines in SSc [56]. They found that miR-21 is notably overexpressed in SSc fibroblasts and that miR-21 expression is regulated by TGFβ. Furthermore, they showed that Smad7) expression is inversely correlated with miR-21 expression since Smad7 was a direct target of miR-21. The authors also stated that bleomycin-induced skin fibrosis is characterized by increased miR-21 expression and could benefit from using bortezomib, which restores miR-21 and Smad7 levels [56]. In our previous study, we demonstrated the elevated expression of miR-21 in serum samples of 50% of a cohort of SSc patients. AUC for miR-21 was estimated at 0.634 (95% CI [0.479–0.790], *p* = 0.147) with 64.7% and 64.3% sensitivity and specificity, respectively. We speculated that the upregulation of miR-21 could be involved in disease pathogenesis [34].

A systematic review on overlapping miRNA signatures in SSc and idiopathic pulmonary fibrosis (IPF), including miR-21, was conducted by Bagnato et al. Since miR-21 exerts putative functions impacting the extracellular matrix, collagen expression, and Smad7 expression, the authors summarized the data on miR-21 and SSc fibrosis [57,58]. The clinical relevance of miR-21 in IPF patients was confirmed by many studies [51,59,60]. It was shown that IPF patients with a rapidly progressive disease have increased levels of miR-21-5p. Furthermore, Li et al. proved the association between miR-21 and worsening FVC and imaging features, and Liu et al. confirmed that miR-21 expression is upregulated in IPF patients’ lungs by alveolar type 2 cells [61,62]. It is important to note that lung fibrogenesis depends on the balance of miR-21 and miR-29, as they have opposite functions. Both miRNAs can regulate the cell cycle of the fibroblasts by regulating cellular proliferation and apoptosis, as well as key functions such as collagen synthesis and breakdown, the transformation of fibroblasts into myofibroblasts, etc. [57].

Furthermore, both miRNAs and their ratio could be used as candidate biomarkers for fibrosis or end-organ damage in SSc. However, it is essential to interpret their levels and relationships carefully. In line with this, in fibroblasts of PF lungs and SSc fibrotic skin, the expression of miR-21 is overexpressed, unlike the downregulated miR-29 [17,61]. Therefore, the association between miR-21-5p elevation and elevated expression of mesenchymal differentiation markers in skin fibroblasts derived from SSc patients is not surprising [17].

miR-21 is able to induce integrin expression that allows the release of TGFβ from its latency associated peptide (LAP) and its binding to its receptors, leading to the activation of Smad3 and stimulating collagen synthesis and α-SMA expression. On the other hand, Smad2 and Smad3 are the primary regulators for miR-21 and miR-29 induction, although they act differently. Smad2 is a negative regulator of miR-21 [57,63]. In contrast, the overexpression of miR-21 blocks the inhibitory effect of Smad7 and Smad3. Additionally, TGF-β is an inducer of miR-21 expression [64].

Wuttge et al. also systematically explored the role of different miRNAs in SSc-associated PAH, where miR-21 was upregulated in SSc with PAH [65]. This finding could be explained as a result of both vascular cellular abnormalities and cardiac-related physiological changes in patients with SSc-associated pulmonary arterial hypertension (SSc-aPAH). Furthermore, the overexpression of mR-21 induced by TGFb reflects the profibrotic activity through the downregulation of Smad7 [29]. Additionally, pulmonary vascular hypoxia also increases the expression of miR-21 [66]. Molecular mechanisms of miR-21 include controlling multiple target genes for PH, such as BMP receptor 2 and hypoxic reprogramming, reflecting the pleiotropic effects of miR-21 in PAH [67]. Analyses also showed that the plasma levels of miR-21-5p combined with miR-20-5p or miR-203a-3p showed the strongest difference between the patient groups [65]. It was also demonstrated that some viral infections, such as HCMV and HHV-6, were capable of modulating the miRNA profile, including miR-21 expression, often more than that documented in SSc, possibly encouraging pathways related to SSc pathogenesis [68].

#### 3.1.4. miR-155

miR-155 was found to be upregulated in fibrotic-related disorders. It has been shown that miR-155 expression is higher in skin biopsies from patients with either a diffuse or limited SSc [69]. Deregulated expression of miR-155 was found in patients with SSc as well as in patients with very early diffuse SSc (VEDOSS) compared to healthy controls [70]. The activation of NLPR-3 inflammasome and subsequent IL-1β signaling mediates miR-155 overexpression in an autocrine mechanism [71,72]. The authors demonstrated that miR-155 expression was upregulated in skin and lung fibroblasts from SSc patients [72]. As abovementioned, miR-155 has been found to regulate lung fibrosis in SSc. The authors observed that miR-155 expression in lung fibroblasts and blood from SSc patients with interstitial lung disease (ILD) correlated with the severity and progression of the lung involvement as compared to the experimental model of miR-155 knock-out mice. Thus, miR-155 could serve as a potential therapeutic target in SSc-ILD [16].

#### 3.1.5. miR-204 and miR-210

Dimitry et al. found that the expression levels of miR-204 in peripheral blood were downregulated and those of miR-210 were upregulated in patients with PAH compared to healthy donors, where miR-210 showed highly significant differences between the PAH groups, including SSc-PAH, idiopathic PAH, and schistosomiasis-associated PAH. Thus, miR-210 could be used as a diagnostic biomarker for SSc-PAH [73].

#### 3.1.6. miR-145 Cluster

It has been found that in response to TGFβ1 microRNA-145-5p (miR-145) exhibits a profibrotic effect by mediating the fibroblast-to-myofibroblast transition [74]. A myofibroblast synthesis marker is human xylosyltransferase-I (XT-I) encoded by the XYLT1 gene, and serum XT activity has been detected in patients with SSc. Interestingly, Ly et al. found that the TGF-β1-induced overexpression of miR-145 in dermal and SSc fibroblasts stimulates cellular XYLT1 expression and XT activity via the downregulation of transcription factor Kruppel-like factor 4 (KLF4). The inhibitory effect of KLF4 on the XYLT1 gene has been proven experimentally through dose-dependent targeted gene silencing in dermal fibroblasts following TGF-β1 stimulation. Thus, the authors identified a new miR-145/KLF4 profibrotic pathway in SSc [74,75].

#### 3.1.7. miR-196a

Alterations in miR-196a expression levels are found in the serum and skin tissue of patients with localized scleroderma. The downregulation of miR-196a in dermal fibroblasts was associated with the upregulation of type I collagen in vitro, thus suggesting that miR-196a may be a critical epigenetic factor in the fibrosis development. Additionally, patients with lower serum levels of miR-196a are reported to have significantly higher rates of diffuse versus limited cutaneous SSc, higher modified Rodnan skin scores, and more pitting scars compared to patients without. Thus, miR-196a is suggested as a serum marker of disease activity in SSc patients [76,77].

#### 3.1.8. miR-130

Another miRNA reported to have profibrotic effects in SSc is miR-130b. According to Luo et al., miR-130b enhances TGFβ signaling and fibrosis-related gene expression through the direct negative regulation of peroxisome proliferator-activated receptor γ (PRARγ) [78]. The PRARγ receptor is known for its regulation of profibrotic responses by revoking TGFβ-stimulation of collagen synthesis, myofibroblast transdifferentiation, and the Smad intracellular signal transduction pathway [79]. miR-130b expression was found to be increased in human SSc skin biopsies and fibroblasts, as well as in the skin fibrosis model, whereas levels of PRARγ were decreased [78,80].

#### 3.1.9. miR-92 and miR-146a

The expression of miR-92a is elevated in the circulation and skin fibroblasts of SSc patients, and its levels correlated with the presence of vascular abnormalities, such as teleangiectasias [81]. Other miRNAs for which abnormal expression was associated with the occurrence of telangiectasia include miR-146a, whose levels were found to be increased in SSc skin biopsies [82].

#### 3.1.10. miR-126, miR-142-3p, and miR-202-3p

Impaired adaptive angiogenesis is a critical pathological factor in SSc. Although there is an upregulation of the main regulator of angiogenesis and vasculogenesis in SSc—the vascular endothelial growth factor (VEGF)—patients with SSc have progressive loss of capillaries and tissue ischemia. Epigenetic regulation of SSc endothelial cell responses to VEGF has been found by Wang et al. The authors found that the downregulation of miR-126 and its gene (*EGFL7*) in SSc endothelial cells and skin is associated with alterations to VEGF by suppressing its negative regulators—sprouty-related protein-1 (SPRED1) and phosphoinositide-3 kinase regulatory subunit 2 (PIK3R2) [83]. Interestingly, Makino et al. found that serum levels of miR-142-3p could be used as a diagnostic biomarker for SSc as they were significantly higher in patients with SSc compared with the control groups, including patients with scleroderma-spectrum disorder, and correlated with disease severity [84]. Zhou et al. found that the overexpression of miR-202-3p in skin tissues of SSc patients is related to increased collagen deposition in dermal fibroblasts. The authors reported that miR-202-3p negatively regulates the fibrotic process in SSc by targeting *MMP-1* gene [85].

### 3.2. miRNA Alterations in Controlling Adipogenesis in SSc Patients

The characteristic feature of SSc is tissue fibrosis, and it is currently known that adipose tissue homeostasis plays a critical role in its pathogenesis. In recent years, it has been shown that skin fibrosis in SSc develops alongside structural damage of subcutaneous adipose tissue (SAT), including both subcutaneous and dermal white adipose tissue (SWAT and DWAT), as well as the loss of adipocytes, though the exact pathogenic mechanism behind this process is still unknown [86]. Tang et al. described a possible control mechanism of adipogenesis by miRNAs. The authors found upregulated miR-4769-3p expression in the plasma and skin lesions of SSc patients and SAT recovery in SSc mouse models when this miRNA was silent. miR-4769-3p inhibits adipogenesis by negatively regulating the ubiquitin-specific protease-18/voltage-dependent anion channel-2 (USP18/VDAC2) axis in adipogenesis. Thus, miR-4769-3p could be used as a prospective new therapeutic target in SSc [28].

In Table 1, we summarize the available data on miRNAs’ role in SSc.

## 4. Prognostic Role of miRNAs in SSc

Beyond advancing our understanding of disease pathogenesis, miRNAs hold strong potential as diagnostic and prognostic biomarkers and may also serve as targets for the development of precision therapies [87].

Makino et al. [84] reported a noteworthy finding regarding miR-142-3p, demonstrating its significantly elevated expression in the serum of patients with SSc. Notably, these levels were markedly different when compared to individuals with scleroderma spectrum disorders (SSDs), systemic lupus erythematosus (SLE), and dermatomyositis (DM), suggesting that miR-142-3p may serve as a potential diagnostic biomarker to distinguish SSc from related autoimmune conditions [84].

In a study by Izumiya et al. [88], the expression of five members of the let-7 microRNA family—let-7a, let-7d, let-7e, let-7f, and let-7g—was analyzed in the context of PH among SSc patients. Through microarray analysis of skin biopsies from 6 patients without PH and 9 with PH, 32 upregulated and 14 downregulated miRNAs were initially identified. Subsequent qRT-PCR validation confirmed that the aforementioned let-7 family members were significantly dysregulated in patients with PH. Moreover, let-7d and let-7b expression correlated with elevated pulmonary arterial pressure, as measured by echocardiography, indicating their potential as biomarkers for PH severity in SSc [88].

Another important aspect of SSc management is the increased risk of malignancy, particularly breast, lung, and hematological cancers [89]. Dolcino et al. [90] explored the role of epigenetic regulation, focusing on miRNA expression profiles potentially linking SSc and oncogenesis. Serum levels of five microRNAs—miR-21-5p, miR-92a-3p, miR-155-5p, miR-16-5p, and miR-126—were assessed by qPCR in 30 SSc patients and 10 healthy controls. Among these, miR-21-5p, miR-92a-3p, miR-155-5p, and miR-16-5p were found to be significantly dysregulated in SSc patients, while miR-126 levels did not differ significantly. The concurrent upregulation of miR-21-5p, miR-92a-3p, and miR-155-5p in both SSc and cancer-related profiles—with known roles in fibrosis, angiogenesis, and cell proliferation—suggests a possible shared epigenetic pathway predisposing SSc patients to malignancy [90].

Although challenges still exist, the use of miRNAs as biomarkers across a range of diseases continues to be a highly promising area of investigation. With ongoing advancements in molecular techniques and bioinformatics, miRNAs are expected to play an integral role in shaping individualized patient profiles, ultimately enabling more precise and targeted therapeutic strategies. However, limitations such as variability in detection methods, the lack of standardization, and insufficient longitudinal data must be addressed before their widespread clinical application can be realized [91].

## 5. Future Directions and Therapeutic Targets

As our understanding of miRNA biology deepens, the therapeutic landscape for SSc is beginning to shift toward molecularly targeted approaches. miRNAs have emerged not only as regulators of gene expression but also as master modulators of key pathogenic processes in SSc, including fibrosis, vascular remodeling, and immune dysregulation. Future research should focus on systematically mapping miRNA signatures across different disease subtypes, organs, and stages to identify consistent patterns that can be translated into clinical biomarkers. High-throughput sequencing technologies and single-cell RNA analysis may further enable the stratification of patients and the identification of disease-driving miRNAs with high specificity [87].

Therapeutically, miRNAs offer several attractive opportunities. On one hand, antagomiRs—chemically modified antisense oligonucleotides [92]—can be used to inhibit profibrotic and pro-inflammatory miRNAs such as miR-21, miR-155, and miR-130b [93,94].

On the other hand, miRNA mimics may be applied to restore the expression of downregulated, protective miRNAs like miR-29a, miR-196a, or miR-126 [95]. Several animal models have already demonstrated the efficacy of such interventions in reducing collagen deposition, inflammation, and vascular damage [96]. However, challenges related to delivery methods, tissue specificity, off-target effects, and long-term safety must be carefully addressed in preclinical and clinical studies.

In the future, integrated therapeutic strategies combining miRNA-based interventions with conventional immunosuppressants or anti-fibrotic agents may offer synergistic effects in halting or even reversing disease progression. Furthermore, the use of circulating miRNAs as non-invasive biomarkers for treatment response and disease monitoring is an area of growing interest. To fully harness the therapeutic potential of miRNAs in SSc, large-scale, longitudinal, and multicenter studies will be essential. These efforts will pave the way toward personalized medicine approaches that incorporate epigenetic profiling, allowing for the selection of miRNA-targeted therapies tailored to the individual patient′s molecular signature.

## 6. Conclusions

In recent years, studies have proven the essential role of circulating and tissue-specific miRNAs in the pathogenesis of SSc. miRNAs control crucial pathways involved in tissue fibrosis, microangiogenesis, and immunological abnormalities. The stability of miRNAs makes them a possible candidate for diagnosis and prognostic biomarkers, as well as new treatment targets in SSc.

## Figures and Tables

**Table 1:** MicroRNAs in systemic sclerosis.

microRNA	Genomic Location	Target Genes/ Pathways	Expression in SSc	Functional Role	Clinical Significance	References
miR-21	17q23.2	Smad7, TGF-β1, BMP-R2	Upregulated	Promotes fibrosis, inflammation, vascular remodeling	Biomarker & potential therapeutic target	[34,51,52,53,54,55,56,57,58,59,60,61,62,63,64,65,66,67]
miR-29a	7q32.3	COL1A1, COL3A1, TAB1	Downregulated	Anti-fibrotic, promotes apoptosis	Potential therapeutic target, biomarker	[27,30,31,32,33,34,35,36,37,38]
miR-27a-3p	19	SPP1, Gremlin1, TGFβR1	Downregulated	Anti-fibrotic, regulates adipogenesis	Associated with ATA, potential therapeutic target	[47,55,56,57,58,59,60,61,62]
miR-155	21q21.3	SOCS1, NLRP3	Upregulated	Pro-inflammatory, regulates lung fibrosis	Marker of ILD severity, therapeutic target	[16,70,71,72,73]
miR-204	9q21.12	Unknown	Downregulated	Linked with PAH	Diagnostic marker in PAH	[73]
miR-210	11p15.5	EFNA3, E2F3	Upregulated	Linked with hypoxia, PAH	Diagnostic biomarker in SSc-PAH	[73]
miR-145	5q32	KLF4, XYLT1	Upregulated	Profibrotic, myofibroblast transition	Biomarker & therapeutic target	[74,75]
miR-196a	12q13.13	COL1A1	Downregulated	Anti-fibrotic	Marker of disease activity in SSc	[76,77]
miR-130b	22q11.21	PPARγ	Upregulated	Enhances fibrosis via TGFβ signaling	Potential profibrotic marker	[78,79,80]
miR-92a	13q31.3	Unknown	Upregulated	Associated with vascular damage	Linked to telangiectasia	[81]
miR-146a	5q33.3	TRAF6, IRAK1	Upregulated	Modulates inflammation	Associated with vasculopathy	[82]
miR-126	9q34.3	SPRED1, PIK3R2	Downregulated	Regulates angiogenesis	Involved in VEGF signaling defects	[83]
miR-142-3p	17q22	Unknown	Upregulated	Diagnostic biomarker	Correlates with disease severity	[84]
miR-202-3p	10q26.3	MMP-1	Upregulated	Regulates collagen deposition	Antifibrotic potential	[85]
miR-4769-3p	Xp11.3	USP18/VDAC2	Upregulated	Inhibits adipogenesis	New therapeutic target	[28,86]

## Abbreviations

The following abbreviations are used in this manuscript:

SSc	Systemic sclerosis
ECM	extracellular matrix
DNA	deoxyribonucleic acid
RNA	ribonucleic acid
miRNA	microribonucleic acid
DGCR8	DiGeorge Syndrome Critical Region 8
AGO	Argonaute
shRNA	Small hairpin ribonucleic acid
TGF	transforming growth factor
CTGF	connective tissue growth
MMP	matrix metalloproteinase
TIMP	tissue inhibitor of metalloproteinases
ATA	antitopoisomerase antibodies
ACA	anticentromere antibodies
EMT	epithelial-mesenchymal transition
VMP1	vacuole membrane protein 1
NLPR-3	NLR Family Pyrin Domain Containing 3
KLF4	Kruppel-like factor 4

## References

[1] Karsulovic C., Hojman L. (2025). Biomarkers in Systemic Sclerosis. Sclerosis.

[2] Maciejewska M., Sikora M., Maciejewski C., Alda-Malicka R., Czuwara J., Rudnicka L. (2022). Raynaud’s Phenomenon with Focus on Systemic Sclerosis. J. Clin. Med..

[3] Ferri C., Arcangeletti M.C., Caselli E., Zakrzewska K., Maccari C., Calderaro A., D’Accolti M., Soffritti I., Arvia R., Sighinolfi G. (2021). Insights into the knowledge of complex diseases: Environmental infectious/toxic agents as potential etiopathogenetic factors of systemic sclerosis. J. Autoimmun..

[4] Zanin-Silva D.C., Santana-Gonçalves M., Kawashima-Vasconcelos M.Y., Oliveira M.C. (2021). Management of Endothelial Dysfunction in Systemic Sclerosis: Current and Developing Strategies. Front. Med..

[5] Flower V.A., Barratt S.L., Ward S., Pauling J.D. (2019). The Role of Vascular Endothelial Growth Factor in Systemic Sclerosis. Curr. Rheumatol. Rev..

[6] Campitiello R., Soldano S., Gotelli E., Hysa E., Montagna P., Casabella A., Paolino S., Pizzorni C., Sulli A., Smith V. (2024). The intervention of macrophages in progressive fibrosis characterizing systemic sclerosis: A systematic review. Autoimmun. Rev..

[7] Ayers N.B., Sun C.M., Chen S.Y. (2018). Transforming growth factor-β signaling in systemic sclerosis. J. Biomed. Res..

[8] Luo Y., Wang Y., Wang Q., Xiao R., Lu Q. (2013). Systemic sclerosis: Genetics and epigenetics. J. Autoimmun..

[9] Walczyk M., Paradowska-Gorycka A., Olesinska M. (2017). Epigenetics: The Future Direction in Systemic Sclerosis. Scand. J. Immunol..

[10] Mei X., Zhang B., Zhao M., Lu Q. (2022). An update on epigenetic regulation in autoimmune diseases. J. Transl. Autoimmun..

[11] Ciechomska M., van Laar J.M., O’Reilly S. (2014). Emerging role of epigenetics in systemic sclerosis pathogenesis. Genes Immun..

[12] Shumnalieva R., Kachakova D., Shoumnalieva-Ivanova V., Miteva P., Kaneva R., Monov S. (2018). Whole peripheral blood miR-146a and miR-155 expression levels in Systemic lupus erythematosus patients. Acta Reumatol. Port..

[13] Shaikh F.S., Siegel R.J., Srivastava A., Fox D.A., Ahmed S. (2024). Challenges and promise of targeting miRNA in rheumatic diseases: A computational approach to identify miRNA association with cell types, cytokines, and disease mechanisms. Front. Immunol..

[14] Yu J., Tang R., Ding K. (2022). Epigenetic Modifications in the Pathogenesis of Systemic Sclerosis. Int. J. Gen. Med..

[15] Luo Y., Xiao R. (2020). The Epigenetic Regulation of Scleroderma and Its Clinical Application. Adv. Exp. Med. Biol..

[16] Christmann R.B., Wooten A., Sampaio-Barros P., Borges C.L., Carvalho C.R., Kairalla R.A., Feghali-Bostwick C., Ziemek J., Mei Y., Goummih S. (2016). miR-155 in the progression of lung fibrosis in systemic sclerosis. Arthritis Res. Ther..

[17] Zhu H., Luo H., Zuo X. (2013). MicroRNAs: Their involvement in fibrosis pathogenesis and use as diagnostic biomarkers in scleroderma. Exp. Mol. Med..

[18] Ballarino M., Pagano F., Girardi E., Morlando M., Cacchiarelli D., Marchioni M., Proudfoot N.J., Bozzoni I. (2009). Coupled RNA processing and transcription of intergenic primary microRNAs. Mol. Cell. Biol..

[19] Cai X., Hagedorn C.H., Cullen B.R. (2004). Human microRNAs are processed from capped, polyadenylated transcripts that can also function as mRNAs. RNA.

[20] Friedman R.C., Farh K.K., Burge C.B., Bartel D.P. (2009). Bartel Most mammalian mRNAs are conserved targets of microRNAs. Genome Res..

[21] Gregory R.I., Chendrimada T.P., Shiekhattar R. (2006). MicroRNA biogenesis; isolation and characterization of the microprocess or complex. Methods Mol. Biol..

[22] Jing Q., Huang S., Guth S., Zarubin T., Motoyama A., Chen J., Di Padova F., Lin S.C., Gram H., Han J. (2005). Involvement of microRNA in AU-rich elemen-mediated mRNA instability. Cell.

[23] Faller M., Guo F. (2008). MicroRNA biogenesis; there’s more than one way to skin a cat. Biochim. Biophys. Acta.

[24] Luo X., Tsai L.M., Shen N., Yu D. (2010). Evidence for microRNA-mediated regulation in rheumatic diseases. Ann. Rheum. Dis..

[25] Yao Q., Xing Y., Wang Z., Liang J., Lin Q., Huang M., Chen Y., Lin B., Xu X., Chen W. (2020). MiR-16-5p suppresses myofibroblast activation in systemic sclerosis by inhibiting NOTCH signaling. Aging.

[26] Henderson J., Pryzborski S., Stratton R., O’Reilly S. (2021). Wnt antagonist DKK-1 levels in systemic sclerosis are lower in skin but not in blood and are regulated by microRNA33a-3p. Exp. Dermatol..

[27] Wajda A., Walczyk M., Dudek E., Stypińska B., Lewandowska A., Romanowska-Próchnicka K., Chojnowski M., Olesińska M., Paradowska-Gorycka A. (2022). Serum microRNAs in Systemic Sclerosis, Associations with Digital Vasculopathy and Lung Involvement. Int. J. Mol. Sci..

[28] Tang B., Yu J., Tang R., He X., Liu J., Liu L., Song Z., Shi Y., Zeng Z., Zhan Y. (2024). MiR-4769-3p suppresses adipogenesis in systemic sclerosis by negatively regulating the USP18/VDAC2 pathway. Iscience.

[29] Zhu H., Li Y., Qu S., Luo H., Zhou Y., Wang Y., Zhao H., You Y., Xiao X., Zuo X. (2012). MicroRNA expression abnormalities in limited cutaneous scleroderma and diffuse cutaneous scleroderma. J. Clin. Immunol..

[30] Qin W., Chung A.C., Huang X.R., Meng X.M., Hui D.S., Yu C.M., Sung J.J., Lan H.Y. (2011). TGF-β/Smad3 signaling promotes renal fibrosis by inhibiting miR-29. J. Am. Soc. Nephrol..

[31] Xu X., Hong P., Wang Z., Tang Z., Li K. (2021). MicroRNAs in Transforming Growth Factor-Beta Signaling Pathway Associated With Fibrosis Involving Different Systems of the Human Body. Front. Mol. Biosci..

[32] O’Reilly S. (2015). miRNA-29a in systemic sclerosis: A valid target. Autoimmunity.

[33] Maurer B., Stanczyk J., Jüngel A., Akhmetshina A., Trenkmann M., Brock M., Kowal-Bielecka O., Gay R.E., Michel B.A., Distler J.H. (2010). MicroRNA-29, a key regulator of collagen expression in systemic sclerosis. Arthritis Rheum..

[34] Shumnalieva R., Kachakova D., Kaneva R., Kolarov Z., Monov S. (2023). Serum miR-21 and miR-29a expression in systemic sclerosis patients. Clin. Exp. Rheumatol..

[35] Jafarinejad-Farsangi S., Gharibdoost F., Farazmand A., Kavosi H., Jamshidi A., Karimizadeh E., Noorbakhsh F., Mahmoudi M. (2019). MicroRNA-21 and microRNA-29a modulate the expression of collagen in dermal fibroblasts of patients with systemic sclerosis. Autoimmunity.

[36] Kawashita Y., Jinnin M., Makino T., Kajihara I., Makino K., Honda N., Masuguchi S., Fukushima S., Inoue Y., Ihn H. (2011). Circulating miR-29a levels in patients with scleroderma spectrum disorder. J. Dermatol. Sci..

[37] Luo Y., Dong H.Y., Zhang B., Feng Z., Liu Y., Gao Y.Q., Dong M.Q., Li Z.C. (2015). miR-29a-3p attenuates hypoxic pulmonary hypertension by inhibiting pulmonary adventitial fibroblast activation. Hypertension.

[38] Wuttge D.M., Carlsen A.L., Teku G., Steen S.O., Wildt M., Vihinen M., Hesselstrand R., Heegaard N.H. (2015). Specific autoantibody profiles and disease subgroups correlate with circulating micro-RNA in systemic sclerosis. Rheumatology.

[39] Li X., Xu M., Ding L., Tang J. (2019). MiR-27a: A Novel Biomarker and Potential Therapeutic Target in Tumors. J. Cancer.

[40] Duwe L., Munoz-Garrido P., Lewinska M., Lafuente-Barquero J., Satriano L., Høgdall D., Taranta A., Nielsen B.S., Ghazal A., Matter M.S. (2023). MicroRNA-27a-3p targets FoxO signalling to induce tumour-like phenotypes in bile duct cells. J. Hepatol..

[41] Mozos A., Catasús L., D’Angelo E., Serrano E., Espinosa I., Ferrer I., Pons C., Prat J. (2014). The FOXO1-miR27 tandem regulates myometrial invasion in endometrioid endometrial adenocarcinoma. Hum. Pathol..

[42] Wang T., Xu Z. (2010). miR-27 promotes osteoblast differentiation by modulating Wnt signaling. Biochem. Biophys. Res. Commun..

[43] Bai L., Lin Y., Xie J., Zhang Y., Wang H., Zheng D. (2021). MiR-27b-3p inhibits the progression of renal fibrosis via suppressing STAT1. Hum. Cell.

[44] Cui H., Banerjee S., Xie N., Ge J., Liu R.M., Matalon S., Thannickal V.J., Liu G. (2016). MicroRNA-27a-3p Is a Negative Regulator of Lung Fibrosis by Targeting Myofibroblast Differentiation. Am. J. Respir. Cell Mol. Biol..

[45] Fang F., Huang B., Sun S., Xiao M., Guo J., Yi X., Cai J., Wang Z. (2018). miR-27a inhibits cervical adenocarcinoma progression by downregulating the TGF-βRI signaling pathway. Cell Death Dis..

[46] Graham J.R., Williams C.M., Yang Z. (2014). MicroRNA-27b targets gremlin 1 to modulate fibrotic responses in pulmonary cells. J. Cell Biochem..

[47] Cheng Q., Chen M., Wang H., Chen X., Wu H., Du Y., Xue J. (2022). MicroRNA-27a-3p inhibits lung and skin fibrosis of systemic sclerosis by negatively regulating SPP1. Genomics.

[48] Zeng X., Huang C., Senavirathna L., Wang P., Liu L. (2017). miR-27b inhibits fibroblast activation via targeting TGFβ signaling pathway. BMC Cell Biol..

[49] Lin Q., Gao Z., Alarcon R.M., Ye J., Yun Z. (2009). A role of miR-27 in the regulation of adipogenesis. FEBS J..

[50] Bayati P., Kalantari M., Assarehzadegan M.A., Poormoghim H., Mojtabavi N. (2022). MiR-27a as a diagnostic biomarker and potential therapeutic target in systemic sclerosis. Sci. Rep..

[51] Sekar D., Hairul Islam V.I., Thirugnanasambantham K., Saravanan S. (2014). Relevance of miR-21 in HIV and non-HIV-related lymphomas. Tumour Biol..

[52] Han M., Liu M., Wang Y., Mo Z., Bi X., Liu Z., Fan Y., Chen X., Wu C. (2012). Re-expression of miR-21 contributes to migration and invasion by inducing epithelial–mesenchymal transition consistent with cancer stem cell characteristics in MCF-7 cells. Mol. Cell. Biochem..

[53] Miao C.G., Xiong Y.Y., Yu H., Zhang X.L., Qin M.S., Song T.W., Du C.L. (2015). Critical roles of microRNAs in the pathogenesis of systemic sclerosis: New advances, challenges and potential directions. Int. Immunopharmacol..

[54] Henry T.W., Mendoza F.A., Jimenez S.A. (2019). Role of microRNA in the pathogenesis of systemic sclerosis tissue fibrosis and vasculopathy. Autoimmun. Rev..

[55] Park J.S., Kim C., Choi J., Jeong H.Y., Moon Y.M., Kang H., Lee E.K., Cho M.L., Park S.H. (2024). MicroRNA-21a-5p inhibition alleviates systemic sclerosis by targeting STAT3 signaling. J. Transl. Med..

[56] Zhu H., Luo H., Li Y., Zhou Y., Jiang Y., Chai J., Xiao X., You Y., Zuo X. (2013). MicroRNA-21 in scleroderma fibrosis and its function in TGF-β-regulated fibrosis-related genes expression. J. Clin. Immunol..

[57] Bagnato G., Roberts W.N., Roman J., Gangemi S. (2017). A systematic review of overlapping microRNA patterns in systemic sclerosis and idiopathic pulmonary fibrosis. Eur. Respir. Rev..

[58] Yamada M., Kubo H., Ota C., Takahashi T., Tando Y., Suzuki T., Fujino N., Makiguchi T., Takagi K., Suzuki T. (2013). The increase of microRNA-21 during lung fibrosis and its contribution to epithelial–mesenchymal transition in pulmonary epithelial cells. Respir. Res..

[59] Yang G., Yang L., Wang W., Wang J., Wang J., Xu Z. (2015). Discovery and validation of extracellular/circulating microRNAs during idiopathic pulmonary fibrosis disease progression. Gene.

[60] Montgomery R.L., Yu G., Latimer P.A., Stack C., Robinson K., Dalby C.M., Kaminski N., van Rooij E. (2014). MicroRNA mimicry blocks pulmonary fibrosis. EMBO Mol. Med..

[61] Li P., Zhao G.Q., Chen T.F., Chang J.X., Wang H.Q., Chen S.S., Zhang G.J. (2013). Serum miR-21 and miR-155 expression in idiopathic pulmonary fibrosis. J. Asthma.

[62] Liu G., Friggeri A., Yang Y., Milosevic J., Ding Q., Thannickal V.J., Kaminski N., Abraham E. (2010). miR-21 mediates fibrogenic activation of pulmonary fibroblasts and lung fibrosis. J. Exp. Med..

[63] Zhong X., Chung A.C., Chen H.Y., Meng X.M., Lan H.Y. (2011). Smad3-mediated upregulation of miR-21 promotes renal fibrosis. J. Am. Soc. Nephrol..

[64] García R., Nistal J.F., Merino D., Price N.L., Fernández-Hernando C., Beaumont J., González A., Hurlé M.A., Villar A.V. (2015). p-SMAD2/3 and DICER promote pre-miR-21 processing during pressure overload-associated myocardial remodeling. Biochim. Biophys. Acta.

[65] Wuttge D.M., Carlsen A.L., Teku G., Wildt M., Rådegran G., Vihinen M., Heegaard N.H.H., Hesselstrand R. (2021). Circulating plasma microRNAs in systemic sclerosis-associated pulmonary arterial hypertension. Rheumatology.

[66] Grant J.S., White K., MacLean M.R., Baker A.H. (2013). MicroRNAs in pulmonary arterial remodeling. Cell Mol. Life Sci..

[67] Negi V., Chan S.Y. (2017). Discerning functional hierarchies of microRNAs in pulmonary hypertension. JCI Insight.

[68] Soffritti I., D’Accolti M., Bini F., Mazziga E., Di Luca D., Maccari C., Arcangeletti M.C., Caselli E. (2024). Virus-Induced MicroRNA Modulation and Systemic Sclerosis Disease. Biomedicines.

[69] Yan Q., Chen J., Li W., Bao C., Fu Q. (2016). Targeting miR-155 to Treat Experimental Scleroderma. Sci. Rep..

[70] Alivernini S., Bosello S.L., De Luca G., Canestri S., Di Mario C., Gigante M.R., Tolusso B., Ferraccioli G. (2014). A3.21 MicroRNA-34a and microRNA-155 in Systemic Sclerosis: Possible epigenetic biomarkers of endothelial dysfunction in VEDOSS and long-standing disease. Ann. Rheum. Dis..

[71] Artlett C.M., Sassi-Gaha S., Hope J.L., Feghali-Bostwick C.A., Katsikis P.D. (2017). Mir-155 is overexpressed in systemic sclerosis fibroblasts and is required for NLRP3 inflammasome-mediated collagen synthesis during fibrosis. Arthritis Res. Ther..

[72] Eissa M.G., Artlett C.M. (2019). The MicroRNA miR-155 Is Essential in Fibrosis. Non-Coding RNA.

[73] Dimitry M.O., Amin Y.M., ElKorashy R.I., Raslan H.M., Kamel S.A., Hassan E.M., Yousef R.N., Awadallah E.A. (2024). Role and predictive value of microRNAs 204 and 210 in the diagnosis of pulmonary arterial hypertension and the distinction between idiopathic, systemic sclerosis, and schistosomiasis-associated pulmonary arterial hypertension. Egypt. J. Bronchol..

[74] Ly T.D., Riedel L., Fischer B., Schmidt V., Hendig D., Distler J., Kuhn J., Knabbe C., Faust I. (2020). microRNA-145 mediates xylosyltransferase-I induction in myofibroblasts via suppression of transcription factor KLF4. Biochem. Biophys. Res. Commun..

[75] Ly T.D., Kleine A., Plümers R., Fischer B., Schmidt V., Hendig D., Distler J.H.W., Kuhn J., Knabbe C., Faust I. (2021). Cytokine-mediated induction of human xylosyltransferase-I in systemic sclerosis skin fibroblasts. Biochem. Biophys. Res. Commun..

[76] Makino T., Jinnin M., Etoh M., Yamane K., Kajihara I., Makino K., Ichihara A., Igata T., Sakai K., Fukushima S. (2014). Down-regulation of microRNA-196a in the sera and involved skin of localized scleroderma patients. Eur. J. Dermatol..

[77] Honda N., Jinnin M., Kajihara I., Makino T., Makino K., Masuguchi S., Fukushima S., Okamoto Y., Hasegawa M., Fujimoto M. (2012). TGF-β-mediated downregulation of microRNA-196a contributes to the constitutive upregulated type I collagen expression in scleroderma dermal fibroblasts. J. Immunol..

[78] Luo H., Zhu H., Zhou B., Xiao X., Zuo X. (2015). MicroRNA-130b regulates scleroderma fibrosis by targeting peroxisome proliferator-activated receptor γ. Mod. Rheumatol..

[79] Ghosh A.K., Bhattacharyya S., Lakos G., Chen S.J., Mori Y., Varga J. (2004). Disruption of transforming growth factor beta signaling and profibrotic responses in normal skin fibroblasts by peroxisome proliferator-activated receptor gamma. Arthritis Rheum..

[80] Li Y., Huang J., Guo M., Zuo X. (2015). MicroRNAs Regulating Signaling Pathways: Potential Biomarkers in Systemic Sclerosis. Genomics Proteom. Bioinform..

[81] Sing T., Jinnin M., Yamane K., Honda N., Makino K., Kajihara I., Makino T., Sakai K., Masuguchi S., Fukushima S. (2012). microRNA-92a expression in the sera and dermal fibroblasts increases in patients with scleroderma. Rheumatology.

[82] Sakoguchi A., Jinnin M., Makino T., Kajihara I., Makino K., Honda N., Nakayama W., Inoue K., Fukushima S., Ihn H. (2013). The miR-146a rs2910164 C/G polymorphism is associated with telangiectasia in systemic sclerosis. Clin. Exp. Dermatol..

[83] Wang Y., Sun J., Kahaleh B. (2021). Epigenetic down-regulation of microRNA-126 in scleroderma endothelial cells is associated with impaired responses to VEGF and defective angiogenesis. J. Cell Mol. Med..

[84] Makino K., Jinnin M., Kajihara I., Honda N., Sakai K., Masuguchi S., Fukushima S., Inoue Y., Ihn H. (2012). Circulating miR-142-3p levels in patients with systemic sclerosis. Clin. Exp. Dermatol..

[85] Zhou B., Zhu H., Luo H., Gao S., Dai X., Li Y., Zuo X. (2017). MicroRNA-202-3p regulates scleroderma fibrosis by targeting matrix metalloproteinase 1. Biomed. Pharmacother..

[86] Varga J., Marangoni R.G. (2017). Systemic sclerosis in 2016: Dermal white adipose tissue implicated in SSc pathogenesis. Nat. Rev. Rheumatol..

[87] Szabo I., Muntean L., Crisan T., Rednic V., Sirbe C., Rednic S. (2021). Novel Concepts in Systemic Sclerosis Pathogenesis: Role for miRNAs. Biomedicines.

[88] Izumiya Y., Jinnn M., Kimura Y., Wang Z., Onoue Y., Hanatani S., Araki S., Ihn H., Ogawa H. (2015). Expression of Let-7 family microRNAs in skin correlates negatively with severity of pulmonary hypertension in patients with systemic scleroderma. Int. J. Cardiol. Heart Vasc..

[89] Hashimoto Y., Akiyama Y., Yuasa Y. (2013). Multiple-to-Multiple Relationships between MicroRNAs and Target Genes in Gastric Cancer. PLoS ONE.

[90] Dolcino M., Pelosi A., Fiore P.F., Patuzzo G., Tinazzi E., Lunardi C., Puccetti A. (2018). Gene Profiling in Patients with Systemic Sclerosis Reveals the Presence of Oncogenic Gene Signatures. Front. Immunol..

[91] Condrat C.E., Thompson D.C., Barbu M.G., Bugnar O.L., Boboc A., Cretoiu D., Suciu N., Cretoiu S.M., Voinea S.C. (2020). miRNAs as Biomarkers in Disease: Latest Findings Regarding Their Role in Diagnosis and Prognosis. Cells.

[92] Lima J.F., Cerqueira L., Figueiredo C., Oliveira C., Azevedo N.F. (2018). Anti-miRNA oligonucleotides: A comprehensive guide for design. RNA Biol..

[93] Pagoni M., Cava C., Sideris D.C., Avgeris M., Zoumpourlis V., Michalopoulos I., Drakoulis N. (2023). miRNA-Based Technologies in Cancer Therapy. J. Pers. Med..

[94] Cerro-Herreros E., González-Martínez I., Moreno N., Espinosa-Espinosa J., Fernández-Costa J.M., Colom-Rodrigo A., Overby S.J., Seoane-Miraz D., Poyatos-García J., Vilchez J.J. (2021). Preclinical characterization of antagomiR-218 as a potential treatment for myotonic dystrophy. Mol. Ther.-Nucleic Acids.

[95] Guo B., Gu J., Zhuang T., Zhang J., Fan C., Li Y., Zhao M., Chen R., Wang R., Kong Y. (2025). MicroRNA-126: From biology to therapeutics. Biomed. Pharmacother..

[96] Greene M.A., Worley G.A., Udoka A.N.S., Powell R.R., Bruce T., Klotz J.L., Bridges W.C., Duckett S.K. (2023). Use of AgomiR and AntagomiR technologies to alter satellite cell proliferation in vitro, miRNA expression, and muscle fiber hypertrophy in intrauterine growth-restricted lambs. Front. Mol. Biosci..

